# Spatial pattern of adaptive and neutral genetic diversity across different biomes in the lesser anteater (*Tamandua tetradactyla*)

**DOI:** 10.1002/ece3.1656

**Published:** 2015-10-15

**Authors:** Camila L. Clozato, Camila J. Mazzoni, Nadia Moraes‐Barros, João S. Morgante, Simone Sommer

**Affiliations:** ^1^ Laboratório de Biologia Evolutiva e Conservação de VertebradosDepartamento de Genética e Biologia EvolutivaInstituto de BiociênciasUniversidade de São PauloR. do Matão, 27705508‐090São PauloBrasil; ^2^ Leibniz‐Institute for Zoo and Wildlife Research (IZW)Evolutionary GeneticsAlfred‐ Kowalke‐Straße 17D‐10315BerlinGermany; ^3^ Berlin Center for Genomics in Biodiversity Research (BeGenDiv)Koenigin‐Luise‐Straße. 6‐8D‐14195BerlinGermany; ^4^ CIBIOCentro de Investigação em Biodiversidade e Recursos GenéticosInBio Laboratório AssociadoUniversidade do PortoR. Padre Armando Quintas4485‐661VairãoPortugal; ^5^ Evolutionary Ecology and Conservation GenomicsUniversity of UlmAlbert‐Einstein Strasse 11D‐89069UlmGermany

**Keywords:** Brazil, *DRB*, major histocompatibility complex, microsatellites, next‐generation sequencing, selection, *Tamandua tetradactyla*

## Abstract

The genes of the major histocompatibility complex (MHC) code for proteins involved in antigen recognition and activation of the adaptive immune response and are thought to be regulated by natural selection, especially due to pathogen‐driven selective pressure. In this study, we investigated the spatial distribution of MHC class II *DRB* exon 2 gene diversity of the lesser anteater (*Tamandua tetradactyla*) across five Brazilian biomes using next‐generation sequencing and compared the MHC pattern with that of neutral markers (microsatellites). We found a noticeable high level of diversity in *DRB* (60 amino acid alleles in 65 individuals) and clear signatures of historical positive selection acting on this gene. Higher allelic richness and proportion of private alleles were found in rain forest biomes, especially Amazon forest, a megadiverse biome, possibly harboring greater pathogen richness as well. Neutral markers, however, showed a similar pattern to *DRB*, demonstrating the strength of demography as an additional force to pathogen‐driven selection in shaping MHC diversity and structure. This is the first characterization and description of diversity of a MHC gene for any member of the magna‐order Xenarthra, one of the basal lineages of placental mammals.

## Introduction

The major histocompatibility complex (MHC) is one of the most important immunogenic systems for infectious disease resistance in vertebrates (Hedrick and Kim 2000). As these genes are highly variable and are thought to play an essential role in the adaptive immune response of vertebrates, they can be useful for investigating the role of natural selection on genetic diversity in wild populations (Bernatchez and Landry 2003).

It has been proposed that pathogen‐mediated selection (PMS) is one of the main driving forces maintaining diversity at MHC loci (Doherty and Zinkernagel 1975; Apanius et al. 1997; Jeffery and Bangham 2000; Bernatchez and Landry 2003) and several hypotheses explaining PMS have been suggested as follows: heterozygote advantage (Doherty and Zinkernagel 1975), rare allele advantage (Slade and McCallum 1992), and fluctuating selection (Hill 1991). All three mechanisms, or a combination of the three, could be the driver of MHC diversity (Hughes and Nei 1988; Takahata and Nei 1990; Apanius et al. 1997). As MHC is known to respond to PMS, the factors that drive pathogen diversity in different environments could also represent important causal predictors for MHC diversity. In fact, contrasting local immunogenetic adaptations of hosts that inhabit habitats with different parasite and pathogen pressure have been reported (e.g.*,* Eizaguirre & Lenz 2010; Lenz et al. 2013; Froeschke and Sommer 2014; Sommer et al. 2014).

Environmental conditions play an important regulating role in the distribution, transmission, and developmental success of parasites and pathogens. Meteorological parameters can influence both the parasite species richness and the intensity of infection in the host species (Mas‐Coma 2008). While temperature is known to be able to increase parasite development rates (Kutz et al. 2005; Hudson 2006), annual precipitation, humidity, and rainfall are important factors explaining diversity of many groups of pathogens, such as bacteria, viruses, fungi, protozoa, and helminths (Appleton and Gouws 1996; Guernier et al. 2004; Froeschke et al. 2010). Climatic effects are also among the main explanation for host species richness (Mittelbach et al. 2007), and it has been observed that it increases with temperature (Austin 1987), and water availability represents a strong predictor for species richness in tropics, subtropics, and temperate zones (Hawkins et al. 2003). Furthermore, several studies demonstrated that parasites diversity is tightly correlated with that of their hosts, not only in terms of species richness, but also the presence of a tight link between their life‐history and ecological traits as well (Poulin and Morand 2000; Kamiya et al. 2014). Thus, parasite/pathogen diversity and pressure along distinct environments should be higher in moister areas harboring higher host species richness (Dunn et al. 2010).

South America is a large territory that includes a vast array of climates, closely associated with vegetation formation. It comprises a tropical region near the equatorial zone, as well as a subtropical region with temperate climates (Fittkau et al. 1969; Sylvestre 2009). The continent shows complex geomorphological patterns (large river plains, e.g.*,* the Amazon basin, and extensive mountain chains, e.g., the Andean Cordillera) (Clapperton 1993). South America harbors the greatest biodiversity on Earth, containing five of the world's biodiversity “hot spots” (Myers et al. 2000). Moreover, South America shows a complex biogeography, composed of several biomes/ecoregions differentiated mostly by vegetation and climate conditions (Morrone 2004, 2006).

The Brazilian flora composition can be divided into six major biomes. The tropical rain forests (Amazon forest and Atlantic forest, average precipitation (AP) of 2600–3600 and 1800–4000 mm/year, respectively), the Brazilian Savanna (Cerrado, AP of 1200–1500 mm/year), the wetlands (Pantanal, AP of 1000–1400 mm/year), the southern grasslands (Campos Sulinos, AP of 1200–1600 mm/year), and the semi‐arid northeast vegetation (Caatinga, AP 300–800 mm/year) (adapted from Veloso et al. 1991; reviewed in Joly et al. 1999; AP retrieved from *Instituto Brasileiro de Geografia e Estatística*, IBGE). Different levels of species richness and endemism are observed between these formations. The Amazon forest is a megadiverse biome (e.g., Hoorn et al. 2010, 2011; Malhado et al. 2013), and the Atlantic forest is considered as a hot spot of biodiversity with high levels of endemism (Myers et al. 2000). Although pathogens species itself have never been fully cataloged and described in each one of the biomes (e.g.*,* Szabó et al. 2007), it is expected that more diverse biomes will carry a more diverse array of pathogens than less diverse ones.

The lesser anteater, *Tamandua tetradactyla* (Linnaeus 1758), is a medium‐sized mammal of the family Myrmecophagidae, order Pilosa. It is part of one of the most ancient lineages of placental mammals, the magna‐order Xenarthra (Murphy et al. 2001), a group that evolved and diversified in South America (Webb 2006). The species has a wide geographic distribution: It occurs in South America east of the Andes, from Venezuela and Trinidad until the north of Argentina, and south of Brazil and Uruguay, in elevations up to 2000 m (Novak 1983; Wetzel 1985; Gardner 2008). Although it has a preference for forested areas, it is also largely found in open grassland savanna‐like areas such as Cerrado, in wetlands such as Pantanal, and in mountain tropical regions (Eisenberg 1989) and transitional forests (Mares et al. 1996). In fact, the species inhabits all major biomes in South America. The lesser anteater is, thus, a suitable model to study the MHC diversity along different habitats (with assumed differences in pathogen diversity) in South America.

In this study, we characterized MHC Class II *DRB* exon 2 diversity for the first time in a member of the magna‐order Xenarthra, in *Tamandua tetradactyla*, and examined the spatial distribution across Brazilian biomes. DRB exon 2 was the chosen because it encodes the functional important antigen‐binding sites mainly involved in extracellular antigen presentation to T cells, and it is, thus, usually highly polymorphic (Bodmer et al. 1990; Hughes and Nei 1990; Hughes and Yeager 1998). Furthermore, DRB exon 2 is probably the most studied MHC gene region in nonmodel organisms, which allows data comparison of genetic diversity across species (Sommer 2005).

Our overall aim was to investigate whether there are different compositions of MHC alleles specific to certain geographic regions which could be indicative of local adaptation to differential pools of pathogens in the landscape, or if MHC alleles are randomly distributed in space, meaning that they are all equally adapted. In the first scenario, we would expect higher levels of genetic diversity in rain forest biomes harboring higher pathogen pressure. Additionally, we compared this pattern of MHC spatial diversity with that of neutral markers, such as microsatellites (after C. L. Clozato, N. Moraes‐Barros, J. S. Morgante unpubl. data), to examine the impact of demographic processes on diversity pattern in the lesser anteater.

## Materials and Methods

### Sampling design

**Figure 1:**
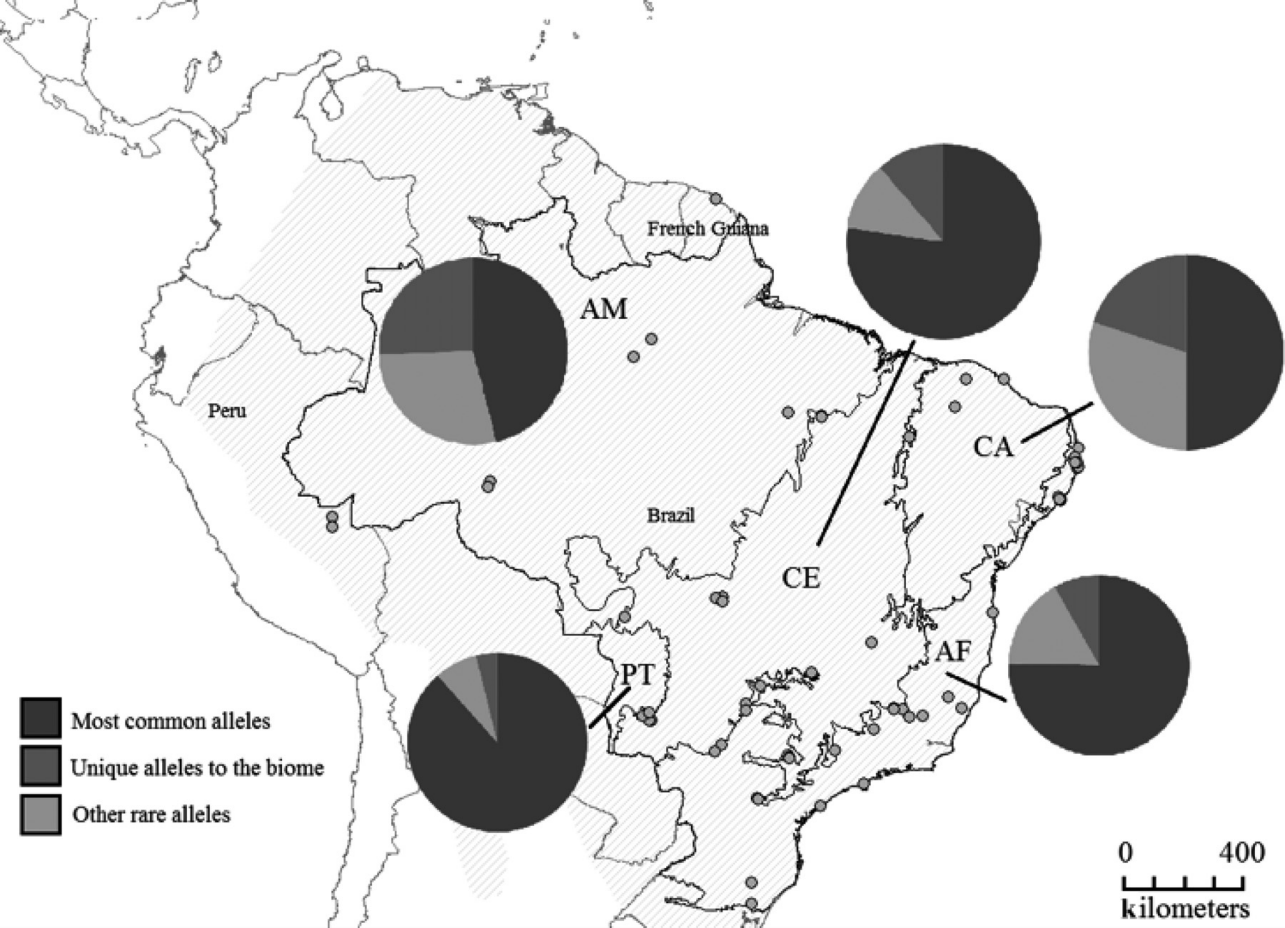
Map of sampling sites of all individuals used in this study across five biomes: Atlantic forest (AF), Amazon forest (AM), Caatinga (CA), Cerrado (CE), and Pantanal (PT). Shaded area throughout South America represents the distribution of *T. tetradactyla*. Solid lines inside Brazil represent biome's limits according to IBGE (http://www.ibge.gov.br/home/), and dark gray dots represent sampling localities. Pie charts indicate the proportion of five most frequent alleles (TateDRB*40, 04, 39b, 01a, and 25), private alleles, and other rare alleles (those detected in two individuals or less) in each biome (see legend).

Genetic samples of *Tamandua tetradactyla* were collected across five Brazilian biomes: Atlantic forest (coastal tropical rain forest, AF, *n = *29), Amazon forest (tropical rain forest, AM, *n* = 12), Caatinga (northeast arid grassland vegetation, CA, *n* = 3), Cerrado (central open grassland vegetation with gallery forests, CE, *n* = 16), and Pantanal (open grassland seasonal wetlands, PT, *n* = 11) between 2006 and 2012 through fieldwork, recovery from road kills, and collaborations with researchers and/or institutions. Additionally, two samples from Peru and one from French Guiana were obtained through donation and assigned to the Amazonian biome. A total of 71 samples were used for this study. Details on the origin of samples and a map of sampling sites are provided in the Appendix and Figure 1, respectively. The biome assignment is based on the geographic coordinates and biomes' definition from the Brazilian Institute of Geography and Statistics (IBGE, http://www.ibge.gov.br/home/) database.

Plucked hair was kept in a dry recipient, and blood and tissue samples were kept in 70% ethanol and kept in 4°C until processed for DNA extraction. Genomic DNA extraction was performed with proteinase K digestion enhanced with DDT 1M, followed by salt precipitation (Sambrook et al. 1989), and stored at −20°C before use. All fieldwork and sample management were performed under the SISBIO/IBAMA authorization for scientific activities number 24001‐5/53695225.

### Preparatory work for the next‐generation sequencing approach: primer design and standardization of amplification efficiency by single‐strand conformation polymorphism (SSCP) and Sanger sequencing

The SSCP technique was used (1) to screen for the best suitable primer pair that catches the most diversity at the target locus, MHC Class II *DRB* exon 2, and minimize the occurrence of artifacts, such as null alleles, and (2) to account for possible differences in the amplification efficiency across loci in the species (Sommer et al. 2013). For this purpose, 10 random samples were used. Tested primers bind to conserved sites of *DRB* intron 1 or exon 2 (forward), and to *DRB* exon 2 or intron 3 (reverse). In total, 59 primer pairs were tested, but only eight pairs yielded satisfactory bands for sequencing. Amplification reactions (PCRs) were conducted in a 20 *μ*L final volume, with 100 ng of template DNA, 0.375 *μ*mol L^−1^ of each primer, 5x HotStar HiFidelity PCR Buffer (including MgSO_4_ and dNTPs mix), 5x Q‐Solution, and 0.5 U of HotStar HiFidelity DNA Polymerase (Qiagen). Thermocycling program consisted of an initial denaturation of 5′ at 94°C, 35 cycles of 1′ at 94°C, 1′ at 53–55°C and 1′ at 72°C, with a final extension of 10′ at 72°C. The chosen primer pair with best yield was JF1 eV (5′‐GAGTGTCATTTYGAGAACGGGACSGAG‐3′) and YML10 (5′‐TCGCCGCTGCACTGTGAACGTCTC‐3′) (Sommer et al. 2013) both binding in the exon 2, amplifying 242 bp. Amplicons derived from successful amplifications were genotyped by SSCP on polyacrylamide gels (Sommer and Tichy 1999). SSCP analysis was performed twice per individual on separate gels using independent PCRs to confirm the banding pattern of all detected alleles. Details about the SSCP preparation, reagents, and electrophoretic conditions are described in Appendix. Single‐strand bands were excised from gel matrix and diluted in distilled water for reamplification in a final volume of 20 *μ*L, 0.375 *μ*mol L^−1^ of each primer, 1.75 *μ*mol L^−1^ dNTP mix, 2.5 of 10× buffer *μ*L, and 0.5 U of Taq polymerase (MP Biomedicals, Santa Ana, CA, USA), using the same program as above. PCR products were purified and sequenced in both directions using BigDye Terminator Cycle Sequencing Kit (Applied Biosystems, Carlsbad, CA, USA) on an ABI PRISM 310 (Applied Biosystems).

Sequence electropherograms were visually inspected using Chromas Lite 1.02 (Technelysium Pty Ltd, Qld, Brisbane, Australia). Alignment and sequence translation were performed with ClustalW algorithm implemented in MEGA 6.0 (Tamura et al. 2013). To check for the histocompatibility nature of the sequences, homology was verified using GenBank database (https://www.ncbi.nlm.nih.gov/genbank/) with the online tool BLASTN (http://www.ncbi.nlm.nih.gov/BLAST). To define a sequence as a putative MHC *DRB* exon 2 allele, the criteria used were its occurrence in at least two independent reactions from the same individuals or detection in at least two distinct individuals. Putative alleles were named according to the nomenclature rules defined in Klein et al. (1990) after confirmation by subsequent pyrosequencing.

### Next‐generation sequencing (NGS) approach

The library preparation for pyrosequencing on a 454 GS Junior Titanium platform (Roche) was performed using fusion primers composed of four parts: (1) adaptor lib A sequence (forward: 5′‐CGTATCGCCTCCCTCGCGCCA‐3′ or reverse: 5′‐CTATGCGCCTTGCCAGCCCGC‐3′), (2) internal library key (TCAG), (3) 10‐base pair‐long barcodes called multiplex identifiers (MIDs) for identification of each sample, and (iv) the sequence of the specific chosen primer pair (forward: JF1 eV or reverse: YML10, see above). A compilation of all fusion primers is available in the Appendix. In total, forward fusion primer was 62 bp long, and reverse was 59 bp. For individual barcoding, eleven forward and ten reverse fusion primers were used. The amount of different MIDs combinations allowed pooling a maximum of 110 tagged samples in each run. To safely assign putative alleles, every individual was separately amplified and sequenced twice using different barcodes (amplicon replicates), following Sommer et al.'s (2013) recommendations to deal with artifacts and allelic dropout. Thus, to genotype all individuals, two independent pyrosequencing runs were necessary. PCR was performed in 25 *μ*L reaction volumes containing 100 ng of template DNA, 0.4 *μ*mol L^−1^ of each fusion primer, 0.2 mmol L^−1^ dNTPs, 2.5 *μ*L FastStart buffer, and 1.25 U FastStart HiFi Polymerase (Roche Diagnostics GmbH, Penzberg, Germany). Thermocycling program consisted of a denaturation step for 2′ at 94°C, followed by 30 cycles consisted of 30″ at 94°C, 30″ at 55°C, and 1′ at 72°C, and a final extension for 7′at 72°C. PCR products were purified through gel band extraction using peqGOLD Gel Extraction Kit (PeqLab, Erlangen, Germany) and quantified by the Quant‐iT PicoGreen dsDNA Assay Kit (Invitrogen Corporation). Subsequently, all amplicons were diluted to 200,000 molecules/*μ*L and pooled. Emulsion PCR, beads recovery, and DNA library enrichment were performed according to the manufacturer's instructions. The enriched library pool was then sequenced on a PicoTiter plate in a 454 GS Junior Titanium (Roche Diagnostics GmbH).

### Quality check of NGS reads and putative allele assignment

Genome Sequencer FLX System Software (Brandfort, CT, USA) was used for initial image and signal processing using the standard amplicon pipeline option. Quality check and filtering steps were performed following the approach and recommendations of Sommer et al. (2013). Briefly, all reads much shorter than expected (~310 bp, including fusion primers and target DNA fragment) were excluded. Reads with incomplete or incorrect MID sequences were removed. Reads with incomplete or incorrect primer regions or <95% bases with Phred quality score Q > 20 were also discarded.

Alignments within individual amplicons were manually performed using the software Geneious Pro v.5.6.5 (Drummond et al. 2012), as none of the alignment software tested managed to correctly place gaps on 454 artificially produced indels. Reads with changes in the reading frame and indels other than 3 bp long (or multiples of 3 bp, corresponding to codons) were excluded due to the biological incompatibility of these features with functional MHC genes. All singleton reads within an amplicon were considered to be artifacts. All remaining reads were clustered based on identity (called clusters), and these consensus sequences were considered hereafter as variants.

The subsequent pipeline used in this study to discriminate “artifacts” from “putative alleles” is illustrated and described in detail in Sommer et al. (2013). Variants within each amplicon were organized based on their frequency, which was considered a criterion for grouping likely putative alleles and probable artifacts because putative alleles were assumed to be in general more frequent than artifacts. At this stage, variants were classified in three categories when comparing them with more frequent variants within the same amplicon: “chimera” (artificial and low‐frequency combination of two parental common variants), “1 bp diff” (i.e., the closest variant among the most frequent ones was only 1 bp apart), and “>1 bp diff.” After this initial variant classification within each amplicon, a second step was performed cross‐checking for correspondence of these variants in the independent amplicon replicate. Artifacts were annotated based on the workflow from Sommer et al. (2013). Variants were classified as “putative allele” if they were present in both individual amplicon replicates and more frequent than all artifacts. A third category defined as “unclassified” was used for variants that were either less frequent than one (or more) artifact(s) or present in only one replicate from an individual, but still could not be considered as artifacts because they were present in other individuals, could not be recognized as possible chimeras, and did not present a very similar (i.e., 1 bp difference) high‐frequency variant. Finally, the remaining variants were classified as artifacts and “putative alleles” by comparing their presence and classification among individuals (Sommer et al. 2013). Whenever variants were more often classified as “unclassified” compared to “putative allele,” they were categorized as “putative alleles with low amplification efficiency.”

### Characterization of MHC class II DRB exon 2 in Tamandua tetradactyla across biomes

Sequences of putative MHC alleles were edited, aligned, and translated using MEGA 6.0 (Tamura et al. 2013). This software was also applied to count the number of variable positions and the mean number of differences between alleles. Nucleotide and amino acid genetic distances were calculated using Kimura‐2‐parameters model of substitution and Poisson‐corrected distance, respectively. For the genetic distance parameter, calculations were performed for the entire data set (all sequences), as well as for each biome separately. The relative rates of nonsynonymous (d*N*) and synonymous (d*S*) base pair substitutions were calculated according to Nei and Gojobori (1986), using Jukes–Cantor correction for multiple hits. These calculations were performed for all sites, for putative antigen‐binding sites (ABS) separately, assuming correspondence to human ABS of HLA‐DR1 molecule (after Brown et al. 1993), and for all sites excluding ABS. The d*N*/d*S* ratios after 1000 bootstrap replicates were compared with an implemented two‐tailed *Z*‐test to test deviation from d*N* = d*S* (positive selection is indicated if d*N* > d*S*, Nei and Kumar 2000). The number of probable loci as product of gene duplications was estimated as the half of the maximum number of alleles for one individual.

The phylogenetic relationships between MHC class II *DRB* exon 2 alleles were investigated by a Bayesian phylogenetic tree obtained by Mr. Bayes vs. 3.2.0 (Ronquist et al. 2012). A mixed model for mixed rates for amino acid with gamma distribution was used. Values of posterior probability were obtained with 300,000 generations and 1000 burn‐in, sampled every 1000 chain to check MCMC convergence. Each terminal (allele) was labeled by its occurrence in each biome. *Dasypus novemcinctus* was used as an out‐group (GenBank sequence XM_004465520.1).

Allelic richness was calculated by a Python script based on the “Multiple random reductions of N” described in Leberg (2002). It performs resampling by taking the minimum number of individuals in each data set, and it extrapolates estimates obtained from small samples to a larger number of individuals. Ten thousand (10000) replicates were used.

Nucleotide diversity was calculated for the entire sequence and for ABS site only with the R package *pegas* (Paradis 2015), considering all MHC alleles and alleles that occur in each biome separately. To envision the sharing of MHC alleles between biomes and individuals, Circus online tool (http://circos.ca/), a method of circular visualization of tables, was used (Krzywinski et al. 2009). Similarly, the evolutionary relationship between MHC alleles, biomes, and individuals was observed through a network taking the required number of mutations between alleles into account using a median‐joining algorithm (Bandelt et al. 1999) implemented in the software Network version 4.612 (http://www.fluxus-engineering.com/sharenet.htm).

We used the *ecodist* package (Goslee and Urban 2007) in R to test the correlation of a matrix of raw individual genetic and geographic distance (Euclidean distances between each pair of individuals measured in log kilometers) applying Mantel tests with 1000 replications (Mantel 1967). We also performed partial Mantel tests to correct for geographic distance considering different biomes as predictor variables for genetic distance.

### Microsatellite data analyses

All samples used in this study were also genotyped at eight microsatellite loci (H5, E12, G3, F1R, B2, C10, A9, and A8). Isolation and characterization of loci are described in Clozato et al. (2014). The forward sequences of microsatellite fragments are deposited in GenBank (Accession Numbers KF746177‐KF746185), and genotyping data of loci for samples used in this study were extracted from C. L. Clozato, N. Moraes‐Barros, J. S. Morgante (unpubl. data). Number of alleles, number of private alleles (averaged across loci and biomes), and observed and expected heterozygosity (*H*_*O*_/*H*_*E*_) were calculated with GenAlEx package 6.41 (Peakall and Smouse 2006). Allelic richness and Mantel tests were also performed for microsatellites as described for *DRB* data.

## Results

### MHC Class II DRB exon 2 diversity and selection pattern in Tamandua tetradactyla

A total of 65 of 71 samples presented consistent 454 pyrosequencing data for further analyses steps. Six samples did not yield enough coverage in one or both replicate amplicons, and were subsequently excluded. After applying the workflow described in the methodology section to classify variants, final data filtering yielded 6402 reads classified as “putative artifacts,” 1194 as “unclassified variants,” and 91,374 as “putative alleles.” For all remaining individuals, the number of reads per amplicon after final data filtering ranged from 110 to 5344, and the number of reads per individual varied between 232 and 10,426.

**Figure 2:**
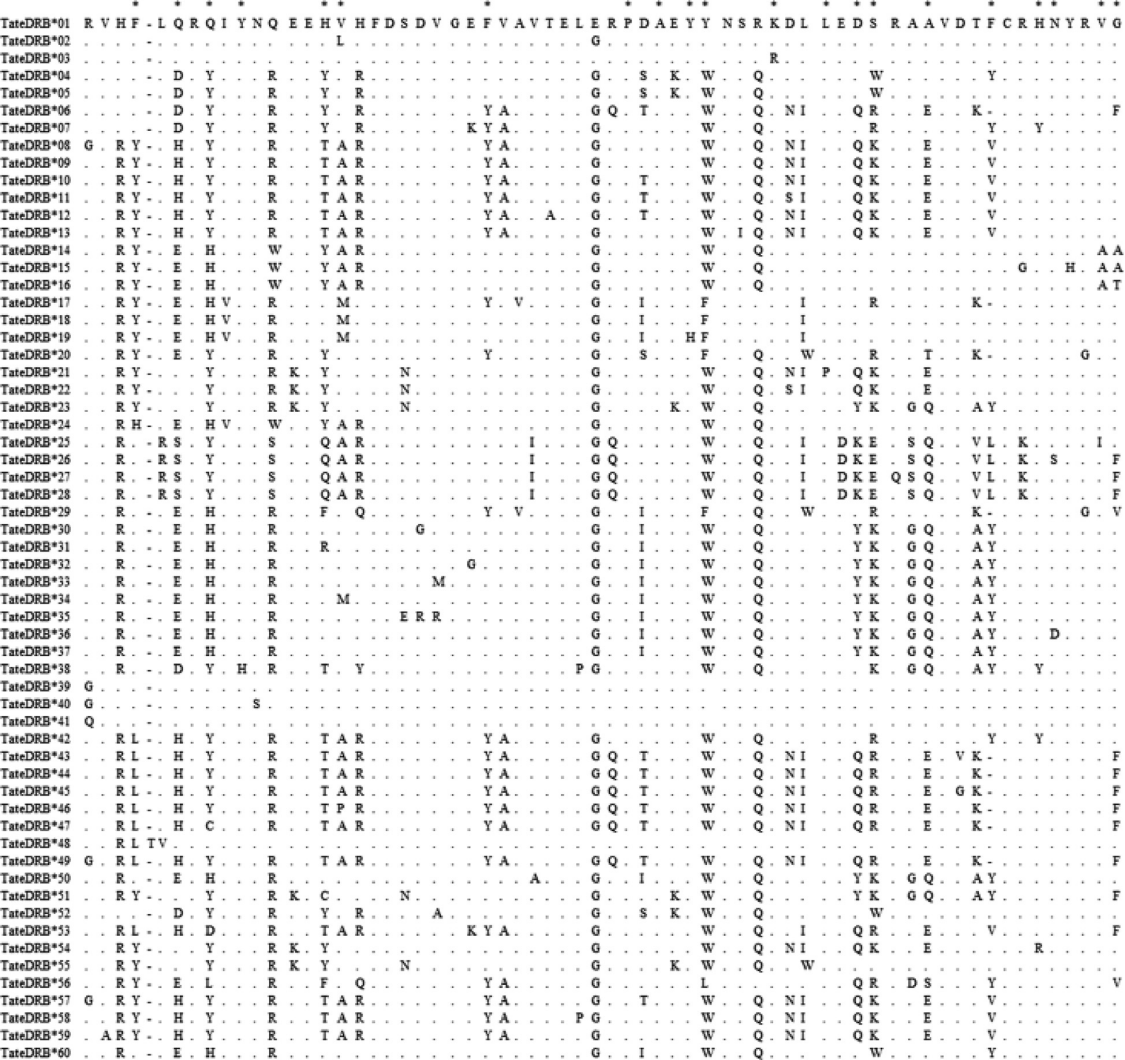
Alignment of *Tamandua tetradactyla* MHC *DRB* exon 2 alleles (amino acids). Dots represent identity to the first sequence, and asterisks represent putative ABS sites inferred after Brown et al. 1993. The first inferred ABS position in the alignment corresponds to position number 26 in *β*‐chain residues of *DRB* gene in the human sequence (Brown et al. 1993).

We validated 70 MHC Class II *DRB* exon 2 alleles in the nucleotide level and 60 alleles in the amino acid level (Table 1, Fig. 2). Average coverage per allele was 1305 reads ranging from 4 to 18,413 (Table 1). Alignment of the nucleotide sequences against GenBank database using BLASTN confirmed the MHC nature of all alleles. *Tamandua tetradactyla* MHC Class II *DRB* allele sequences were deposited in GenBank under the Accession Numbers KP780001 ‐ KP780057. One allele (TateDRB*48) presented a 3‐bp insertion at position 13–15 of the nucleotide alignment, and 13 alleles (TateDRB*06, 17, 20, 29, 43, 44a, 44b, 44c, 44d, 45, 46, 47, and 49) presented a 3‐bp deletion at position 169–171. Both indels represent one single codon (inframe alteration), so the reading frame was unaltered.

**Table 1:** MHC class II *DRB* exon 2 alleles observed in *Tamandua tetradactyla* across five biomes. *N* is the number of individuals that contain a specific allele. Relative frequency of a specific allele in the overall data set (65 individuals). MHC alleles that are similar at the amino acid level but different at the nucleotide level are indicated with “a,” “b,” “c,” and “d.” Coverage refers to overall number of reads for each allele. The total number of validated reads was 91,374

*DRB* allele	*N*	Frequency	Coverage	*DRB* allele	*N*	Frequency	Coverage
TateDRB*01a	21	0.323	7289	TateDRB*34	1	0.015	11
TateDRB*01b	4	0.061	9408	TateDRB*35	1	0.015	6
TateDRB*02	3	0.046	132	TateDRB*36	3	0.046	115
TateDRB*03	14	0.215	6686	TateDRB*37a	2	0.030	143
TateDRB*04	32	0.492	18413	TateDRB*37b	5	0.076	1707
TateDRB*05	9	0.138	1938	TateDRB*37c	2	0.030	43
TateDRB*06	1	0.015	281	TateDRB*38	11	0.169	140
TateDRB*07	2	0.030	58	TateDRB*39a	4	0.061	29
TateDRB*08	2	0.030	63	TateDRB*39b	26	0.400	10568
TateDRB*09	5	0.076	3172	TateDRB*39c	1	0.015	240
TateDRB*10	1	0.015	16	TateDRB*39d	4	0.061	147
TateDRB*11	2	0.030	768	TateDRB*40	37	0.569	971
TateDRB*12	1	0.015	95	TateDRB*41	1	0.015	144
TateDRB*13	1	0.015	464	TateDRB*42	4	0.061	555
TateDRB*14	3	0.046	894	TateDRB*43	7	0.107	1208
TateDRB*15	2	0.030	160	TateDRB*44a	11	0.169	4314
TateDRB*16	2	0.030	15	TateDRB*44b	5	0.076	636
TateDRB*17	7	0.107	74	TateDRB*44c	15	0.230	4095
TateDRB*18a	1	0.015	7	TateDRB*44d	2	0.030	1193
TateDRB*18b	1	0.015	44	TateDRB*45	3	0.046	309
TateDRB*19	1	0.015	4	TateDRB*46	1	0.015	273
TateDRB*20	1	0.015	23	TateDRB*47	2	0.030	39
TateDRB*21	1	0.015	29	TateDRB*48	4	0.061	225
TateDRB*22	1	0.015	24	TateDRB*49	8	0.123	74
TateDRB*23	1	0.015	8	TateDRB*50	1	0.015	4
TateDRB*24	2	0.030	522	TateDRB*51	2	0.030	35
TateDRB*25	19	0.292	8168	TateDRB*52	1	0.015	5
TateDRB*26	1	0.015	6	TateDRB*53	4	0.061	77
TateDRB*27	1	0.015	66	TateDRB*54	1	0.015	4
TateDRB*28	10	0.153	2341	TateDRB*55	1	0.015	4
TateDRB*29	11	0.169	1338	TateDRB*56	2	0.030	7
TateDRB*30	7	0.107	161	TateDRB*57	1	0.015	4
TateDRB*31	2	0.030	450	TateDRB*58	1	0.015	6
TateDRB*32	1	0.015	33	TateDRB*59	1	0.015	4
TateDRB*33	2	0.030	885	TateDRB*60	1	0.015	4

Sixteen of the 70 MHC alleles (22.9%) were frequent and detected in more than 10% of the individuals, whereas more than half (41, 58.5%) of alleles were rare, that is, found in two or less individuals (this definition of rare alleles is considered in all further results). Rare MHC alleles were distributed in all sampling regions (Fig. 1). All MHC alleles (frequent, rare, and private alleles) were confirmed and validated, because they occurred in both independent replicates (Table 1, Fig. 4A).

Number of *DRB* alleles across all individuals varied between two and 13, indicating that at least seven loci were amplified. The general MHC *DRB* diversity features are summarized in Table 2. A total of 75 nucleotide sites were invariable, and 114 were variable. Among these, 35 sites were singletons and 79 sites were parsimony informative. Overall MHC allele sequence diversity was 1.000 ± 0.003, and nucleotide diversity was 0.132 ± 0.004. When considering only nucleotide sequences, all inferred ABS sites were variable in at least one codon position. In translated amino acid sequences, all but two inferred ABS positions were variable (Fig. 2).

**Table 2:** Genetic diversity in the overall *Tamandua tetradactyla* data set and in each biome for MHC class II *DRB* exon 2 and for eight microsatellite loci (*N* = sample size; *H*_*O*_ */H*_*E*_ *= *observed and expected heterozygosity; mean number of alleles and private alleles are averaged across all loci)

Data set	*N*	MHC					Microsatellites		
		*DRB* nucleotide alleles	*DRB* amino acid alleles	Nucleotide alleles per individual	Amino acid sequences		Mean *H*_*O*_ */H*_*E*_	Mean number of alleles	Mean number of private alleles
		(195 bp)	(65 codons)		Variable positions (%)	Mean number of differences (±SE)			
All samples	65	70	60	1–13	53/65 (82)	14.60 (±1.97)	0.38/0.49	2.83 (±0.69)	0.27 (±0.13)
Atlantic forest	29	43	36	1–11	48/65 (74)	14.31 (±1.94)	0.40/0.52	6.00 (±1.55)	0.75 (±0.25)
Amazon forest	9	32	28	3–10	46/65 (71)	14.86 (±2.11)	0.50/0.56	5.13 (±1.30)	0.75 (±0.36)
Cerrado	16	32	29	2–9	45/65 (69)	15.48 (±2.14)	0.36/0.48	5.13 (±1.30)	0.50 (±0.26)
Caatinga	3	15	14	2–5	33/65 (51)	13.12 (±1.88)	0.33/0.47	2.87 (±0.47)	0.13 (±0.25)
Pantanal	8	21	15	4–13	38/65 (58)	15.05 (±2.14)	0.29/0.40	3.5 (±0.92)	0.00 (±0.00)

The d*N*/d*S* ratio was 2.94; that is, a higher value of d*N* than expected for neutral regions was observed, which is compatible with a scenario where this genomic region is under positive selection. This ratio was clearly driven by ABS positions, which held the most part of nonsynonymous substitutions. The two‐tailed *Z*‐test corroborated the hypothesis of positive selection acting on ABS sites (*P*‐value* = *0.035) (Table 3). Estimates of genetic distances between alleles were more pronounced in among amino acid sequences than nucleotide sequences, which points to the functionality of these different alleles.

**Table 3:** The estimated rates (±SE) of d*N* and d*S* substitutions for all sites (65 codons), ABS (21 codons) and non‐ABS (44 codons), and their ratio (d*N/*d*S*) for MHC class II *DRB* exon 2 sequences in *Tamandua tetradactyla*. The probability that d*N* and d*S* are different (null hypothesis of neutrality is d*N = *d*S*) was calculated using a two‐tailed codon based *Z*‐test, with 1000 bootstrap replications to obtain the *P*‐value. Significance at 0.05 is indicated by an asterisk

Positions	d*N*	d*S*	d*N/*d*S*	*P*‐value
All sites	0.306 (±0.081)	0.104 (±0.044)	2.94	0.142
ABS	0.279 (±0.070)	0.138 (±0.058)	2.02	0.035*
Non‐ABS	0.099 (±0.021)	0.143 (±0.041)	0.69	0.948

### Comparison of individual MHC genotypes obtained by the SSCP and NGS approach

**Figure 3:**
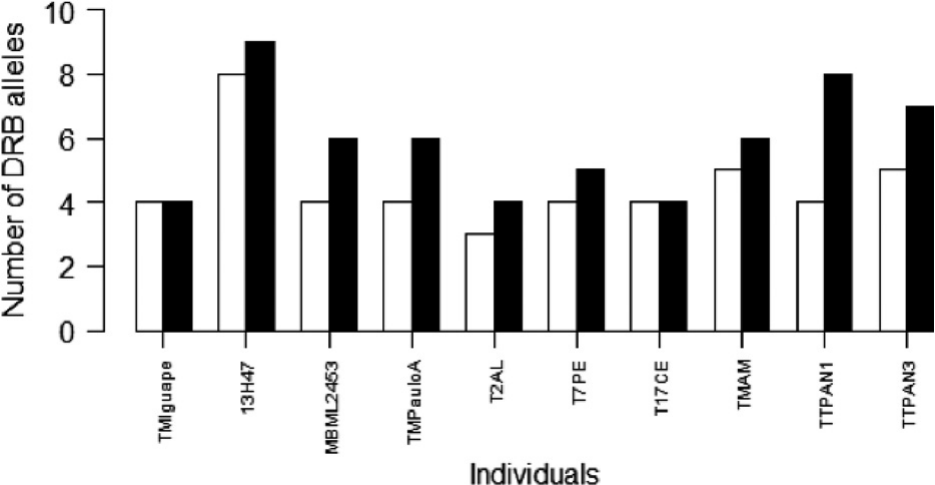
Comparison of number of MHC Class II *DRB* exon 2 putative alleles retrieved for ten individuals through SSCP (white bars) technique and next‐generation sequencing (NGS, black bars). Significantly more MHC alleles per individual were detected by the NGS approach (Student's *t*‐test for paired data* = *3.772, *P*‐value* = *0.004).

A comparison of *DRB* putative alleles retrieved from SSCP and NGS techniques for ten individuals revealed that additional MHC alleles could be detected using NGS in all but two of the samples (Student's *t*‐test for paired data* = *3.772, *P*‐value* = *0.004, Table 4, Fig. 3). SSCP technique was able to detect 25 MHC Class II *DRB* exon 2 alleles in the nucleotide level and 20 alleles in the amino acid level among ten samples analyzed with this approach. In SSCP, the number of different MHC alleles found in one individual varied between three and eight (Table 4), which would indicate the presence of at least four *DRB* loci, an underestimation of the underlying loci diversity if compared to NGS.

**Table 4:** Comparison of putative MHC alleles obtained for ten individuals using the SSCP gel or the 454 pyrosequencing approach (*N*_*1*_ and *N*_*2*_ are the total number of putative alleles found in each individual for SSCP and NGS, respectively). Additional MHC alleles detected by NGS only are marked in bold

Samples	*N*_*1*_	*N*_*2*_	Putative alleles				
TMIguape	4	4	TateDRB*04	TateDRB*40	TateDRB*39d	TateDRB*17	
13H47	8	9	TateDRB*04	TateDRB*44c	TateDRB*44a	TateDRB*39b	TateDRB*38
			TateDRB*01a	TateDRB*43	TateDRB*11	TateDRB*29	
MBML2453	4	6	TateDRB*40	TateDRB*39a	TateDRB*01a	TateDRB*44c	TateDRB*01a
			TateDRB*25				
TMPauloA	4	6	TateDRB*37b	TateDRB*36	TateDRB*30	TateDRB*39b	TateDRB*25
			TateDRB*40				
T2AL	3	4	TateDRB*04	TateDRB*05	TateDRB*29	TateDRB*40	
T7PE	4	5	TateDRB*04	TateDRB*05	TateDRB*40	TateDRB*29	TateDRB*17
T17CE	4	4	TateDRB*40	TateDRB*04	TateDRB*42	TateDRB*53	
TMAM	5	6	TateDRB*04	TateDRB*01a	TateDRB*44a	TateDRB*31	TateDRB*57
			TateDRB*56				
TTPAN1	4	8	TateDRB*40	TateDRB*01b	TateDRB*39c	TateDRB*39d	TateDRB*03
			TateDRB*25	TateDRB*44a	TateDRB*49		
TTPAN3	5	7	TateDRB*40	TateDRB*28	TateDRB*39a	TateDRB*01a	TateDRB*45
			TateDRB*25	TateDRB*39d			

### MHC diversity pattern across biomes

**Figure 4:**
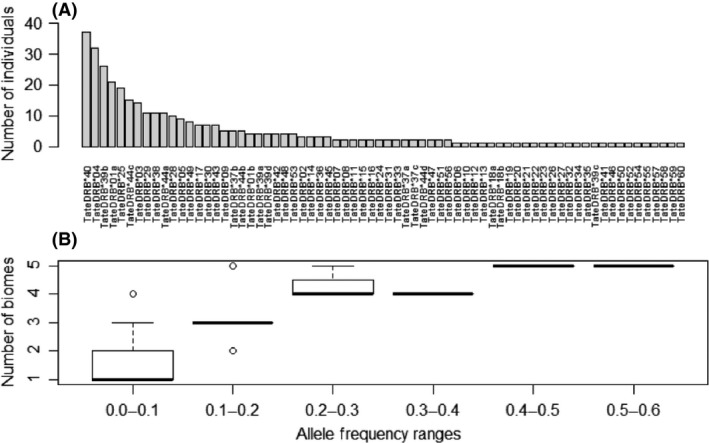
(A) Frequency of MHC class II *DRB* nucleotide alleles in 65 individuals. Sixteen alleles were detected in more than 10% of the individuals, whereas more than half of alleles are found in only one or two individuals, but confirmed and validated because they occur in both independent replicates. (B) Box plots of MHC class II allele frequencies divided in intervals of 0.1 and the number of biomes (1–5) in which they occur.

**Figure 5:**
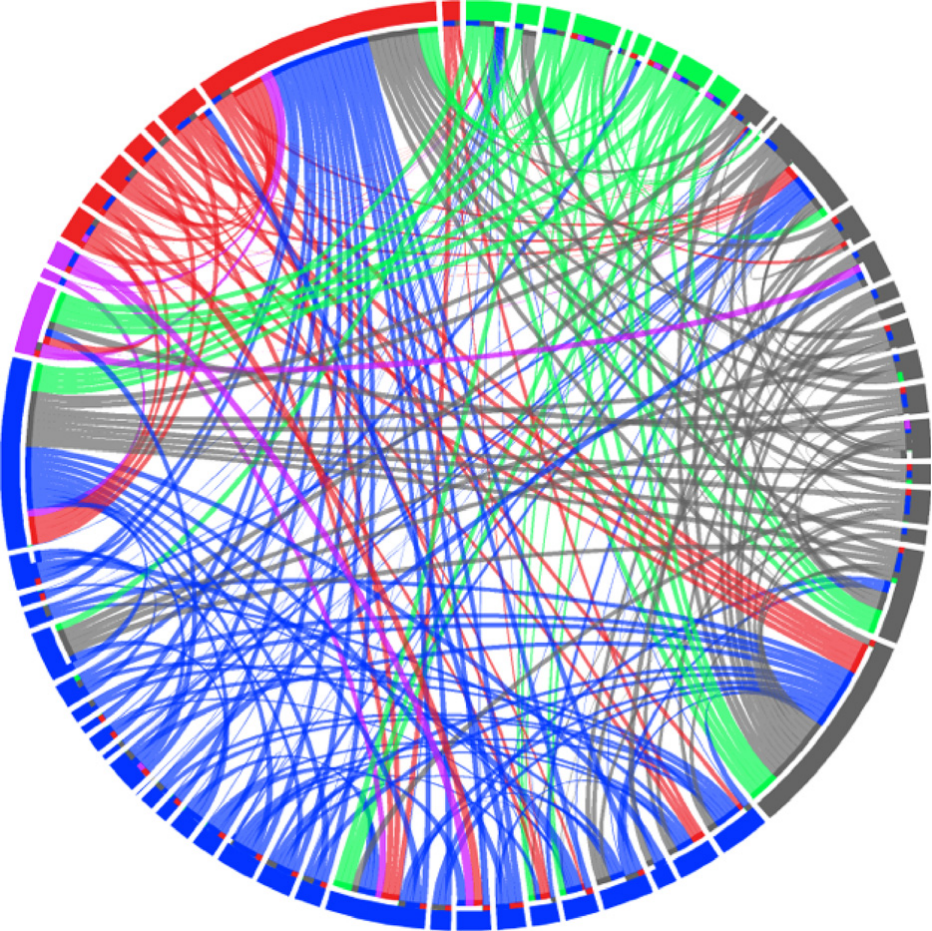
Sharing of MHC class II *DRB* exon 2 alleles among individuals within and between different biomes. Biomes are denoted by outer circle color: Atlantic forest (blue, *n* = 29), Amazon forest (green, *n* = 9), Cerrado (gray, *n* = 16), Caatinga (violet, *n* = 3), and Pantanal (red, *n* = 8). Each ribbon represents one *DRB* amino acid allele (*N* = 60). Each interruption in the outer circle represents a different individual (*N* = 65). Note that most private alleles (present in one biome only) where found in several individuals.

Some MHC alleles were more frequent and common than others (Table 1, Fig. 4). MHC alleles with high or intermediate frequency were widely distributed across the diverse habitats used by the lesser anteater. Alleles present in more than 10% of individuals (Fig. 4A) were distributed in three or more biomes. Some other MHC alleles, less frequent (<10% of individuals) but yet not rare (i.e., present in more than two individuals), were likewise distributed in different biomes (TateDRB*48, 17, 37, 42, 09, 53, 43, and 49). *DRB* alleles with frequencies as high as 0.5 occurred in all five biomes indicating their wide geographic range, whereas alleles with low frequencies (0.1–0.2) occurred in three or less biomes (Figs 4B and 5).

A total of 33 (50.8%) of all *DRB* amino acid alleles were private to single biomes, either present in one individual or shared among individuals of the same biome (Fig. 1). Atlantic forest and Amazon forest presented the highest number of private alleles: 12 and 11 of 33 alleles (36 and 33%, respectively). Cerrado, Caatinga, and Pantanal presented 5, 3, and 2 private alleles (15, 9, 6%), respectively. When considering samples partitioned by biomes, Atlantic forest presented a set of sequences with most variable positions (74%) and Caatinga with the least (51%) (but also the lowest samples size, *n* = 3). The highest mean number of differences between sequences was found in Cerrado (Table 2).

**Figure 6:**
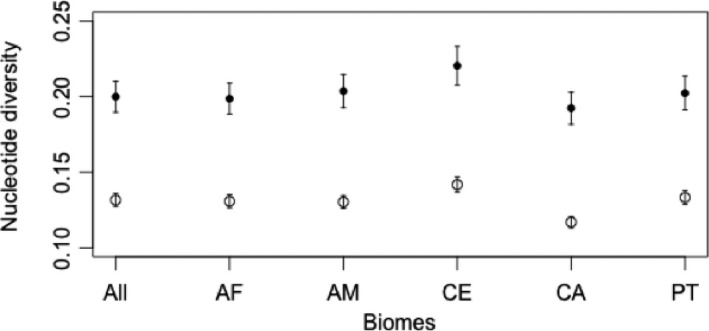
Plot of nucleotide diversity (±standard deviations) for MHC class II DRB allele sequences in the overall sample and per biome. Black dots represent antigen‐binding sites (ABS) and white dots represent all sites.

For the whole sequence of *DRB*, nucleotide diversity ranged between 0.117 (CA) and 0.142 (CE). Nucleotide diversity in ABS ranged between 0.011 (CA) and 0.013 (CE). Again Caatinga's (CA) results in genetic diversity (both nucleotide diversity and variable positions) may be influenced by the low sample size (*n* = 3). Overall, nucleotide diversity inside ABS was consistently higher than the entire sequence, regardless of biomes tested (Fig. 6). In all biome partitions, ABS genetic distance was constantly more divergent than non‐ABS fragments.

**Figure 7:**
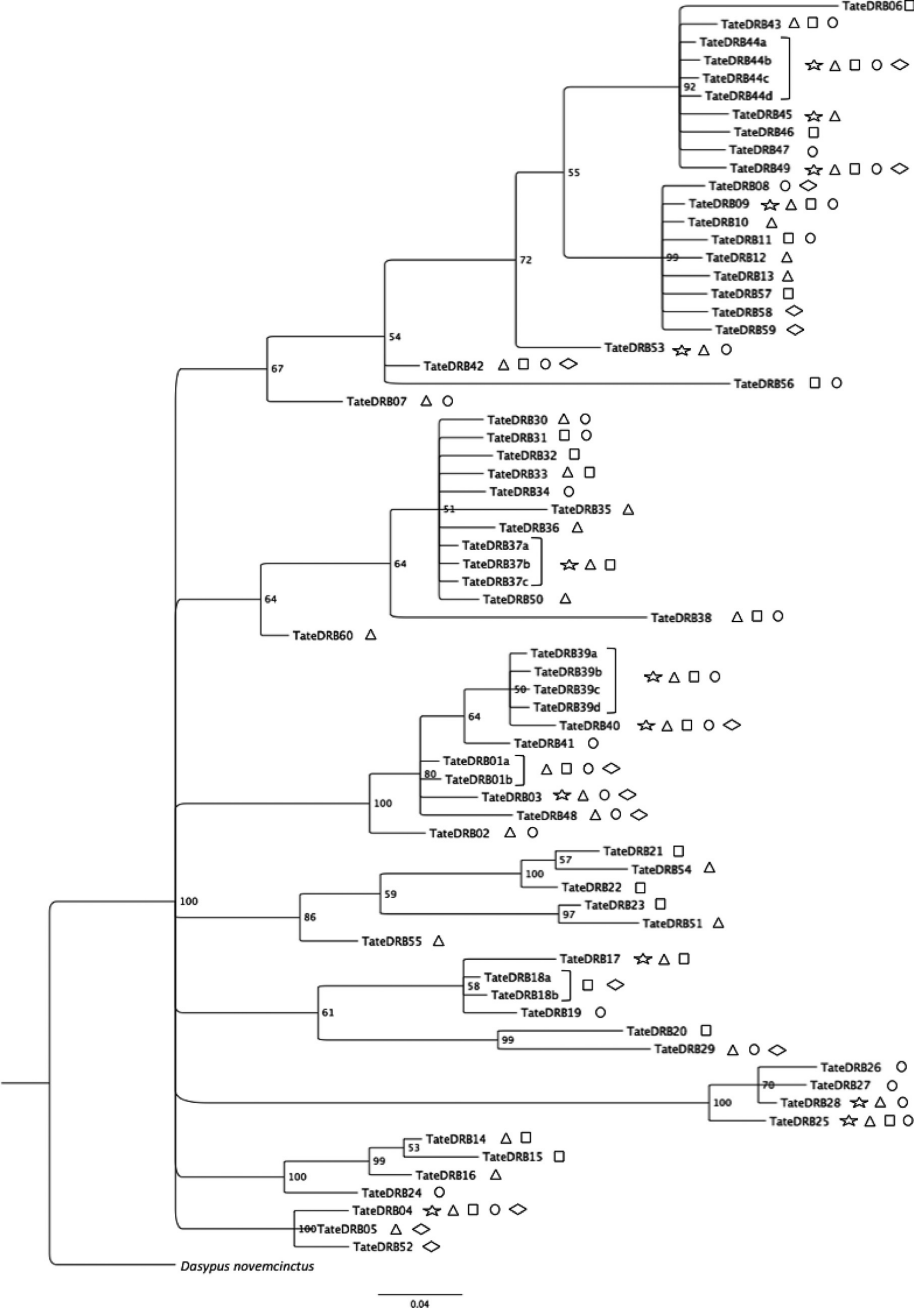
Bayesian phylogenetic tree of MHC Class II *DRB* alleles with values of posterior probabilities for nodes. Terminals are labeled with biomes in which alleles occur: Atlantic forest (triangle), Amazon forest (square), Cerrado (circle), Caatinga (diamond), and Pantanal (star). *Dasypus novemcinctus* was used as out‐group.

**Figure 8:**
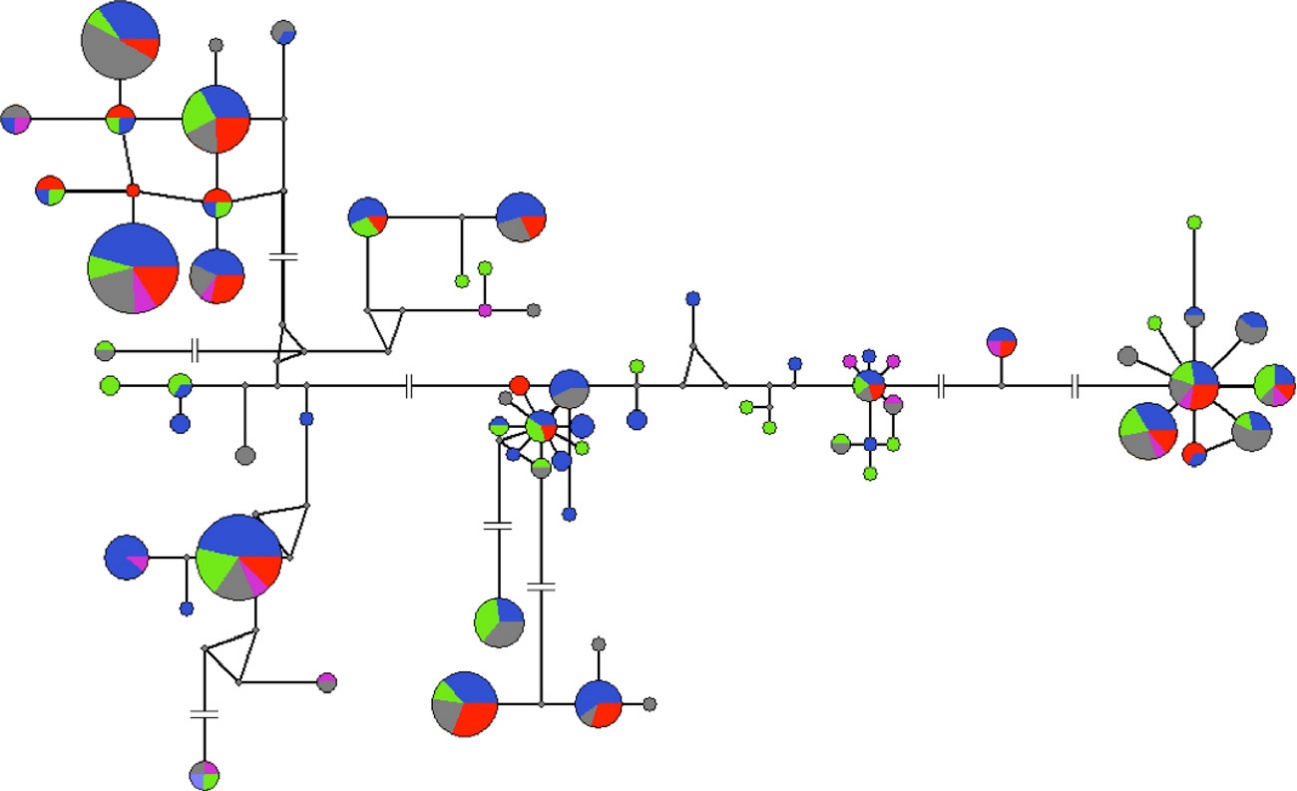
Network of MHC class II *DRB* exon 2 alleles (nucleotide level) with nodes proportional to frequency of individuals carrying the allele, colored by the biomes in which it occurs: Atlantic forest (blue, *n* = 29), Amazon forest (green, *n* = 9), Cerrado (gray, *n* = 16), Caatinga (violet, *n* = 3), and Pantanal (red, *n* = 8). Interruptions in lines represent the presence of more than ten mutations.

Phylogenetic tree of *DRB* alleles resulted in roughly nine major clades, with variable branch lengths reflecting the differences between alleles (Fig. 7). Among nine clades displayed in the phylogeny, seven contain at least one geographically disseminated allele of high or intermediate frequency. Considering that each clade could possibly represent one locus or closely related loci, these seven clades would be composed of one widespread MHC allele (or more) and a few biome‐specific alleles. Some clades are predominantly clusters of MHC alleles specific to biomes AF and AM. There is also constant grouping of alleles between these biomes along the phylogenetic tree and vast allele sharing between them (Figs 5 and 8).

### Comparison of DRB and microsatellite diversity and correlation with geographic distance

**Figure 9:**
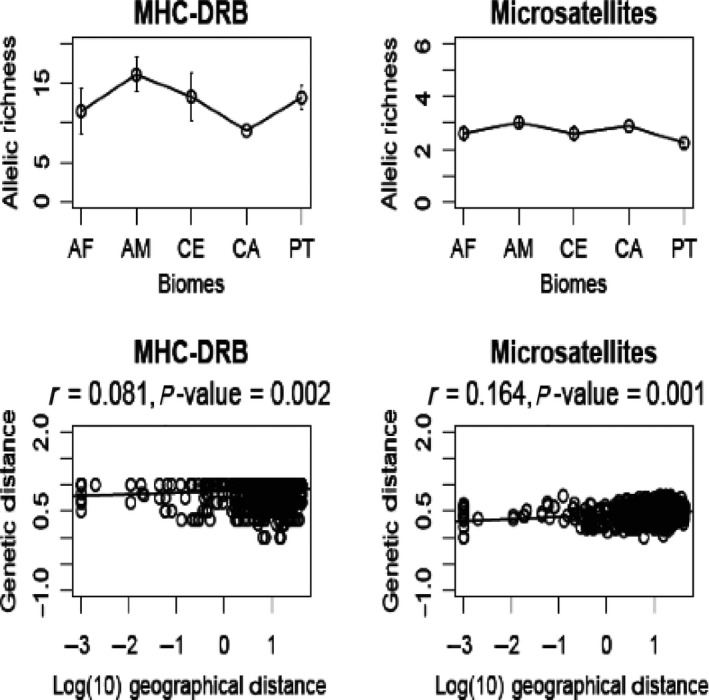
Allelic richness values (±standard deviations) across biomes for MHC class II *DRB* exon 2 and for microsatellite loci (above). Plots are shown after standardization for a minimum of three samples (all biomes). Mantel tests showing the relationship between individual genetic and geographic distances (Log 10) for *DRB* alleles and microsatellite loci, and their respective correlation values (below).

MHC allelic richness corrected for three individuals (all biomes) ranged between 9.0 (CA) and 16.10 (AM) (Fig. 9). For microsatellite data, AM also displayed the highest allelic richness (for a minimum of three samples, allelic richness ranged between 2.3 in PT and 3.0 in AM) (Fig. 9). In all cases, a higher value of allelic richness was found for *DRB* data, although not directly comparable due to different modes of evolution. Even though both data sets indicated higher allelic richness value in Amazon forest, this difference was steeper in *DRB*.

In microsatellites, the highest mean number of microsatellite alleles was observed in the Atlantic forest, while the highest number of private alleles was observed both in Atlantic forest and in Amazon forest. Also, mean *H*_*O*_ */H*_*E*_ was higher in Amazon and Atlantic forests than in other biomes (Table 2).

Positive and significant correlation between genetic and geographic distances was found for both data sets (*DRB* and microsatellites), although the degree of this correlation was much stronger for microsatellites than for *DRB* (*DRB*: *r* = 0.081, *P*‐value* = *0.002; microsatellites: *r* = 0.164, *P*‐value* = *0.001) (Fig. 9). When using a partial Mantel test to correct for the effects of geographic distance (i.e., testing the response of genetic distance to different biomes, representing different environments), a similar positive correlation is observed for both *DRB* and microsatellites (*DRB*:* r* = 0.354 *P*‐value* = *0.001; microsatellites: *r* = 0.351, *P*‐value* = *0.001).

## Discussion

In this study, we examined the MHC Class II *DRB* exon 2 sequence diversity of the lesser anteater for the first time. In fact, this was the first characterization and description of diversity of a MHC gene for any member of the magna‐order Xenarthra, one of the basal lineages of placental mammals, along with Afrotheria (Murphy et al. 2001). Across a wide sampling region, we aimed to investigate whether there are different compositions of MHC alleles specific to certain geographic regions which could be indicative of local adaptation, or whether alleles are randomly distributed in space.

### MHC Class II DRB diversity

We were able to describe 60 *DRB* exon 2 alleles detected in 65 individuals of *Tamandua tetradactyla* on the amino acid level and 70 on the nucleotide level (Table 1). As our sampling strategy covered a broad territorial extension and many different landscapes (referred to as “biomes” in this study), we believe to have reported the major alleles of *DRB* exon 2 across the species distribution.

Considering the amount of samples successfully processed, this is a considerable high level of diversity, even for a locus already expected to be highly variable. This could be the result of two complementary facts. First, the individuals sampled were spread over a vast geographic area, comprising distinct environments. The sampling sites of *Tamandua tetradactyla* were chosen intending to represent the actual geographic distribution of the species along South America (mainly Brazil, which comprises the majority of the continent's territory) (Fig. 1). Therefore, the samples originated from a variety of distinct environments, and the high diversity of *DRB* exon 2 may mirror the species‐wide distribution. Secondly, NGS technique used in this study provided a wider capture of MHC *DRB* alleles. NGS technique has proven to be more sensitive to identify different sequence variants than the SSCP approach (Fig. 3), especially for alleles with low amplification efficiency (Sommer et al. 2013). Other studies have also reported this trend (e.g., Promerová et al. 2012; Sommer et al. 2013), and this may have contributed to the high amount of alleles found for *Tamandua tetradactyla* as well.

Among mammals, different levels of MHC Class II gene diversity have been reported. In nonvolant widely distributed mammals, as many as 36 alleles were described for more than one hundred samples of the European dog *Canis lupus* (Seddon and Ellegren 2002), and 52 alleles were described for 25 samples of the Asiatic lion *Panthera leo* (Sachdev et al. 2005), both using traditional methods (respectively, SSCP and cloning followed by sequencing). On the other hand, 58 *DRB* alleles were described based on 36 samples of *Delomys sublineatus*, a small mammal comprised in a restricted distribution (compared to lesser anteater), using a NGS approach (Sommer et al. 2013). Thus, the technique used to describe the diversity of MHC genes in a species sample set is relevant (along with sample size), and it may contribute to correlations of diversity with the species biology.

The high number of *DRB* alleles found in *T. tetradactyla*, however, could also be related to copy number variation in the species. In an extreme example, one individual (TTPAN7) displayed 13 alleles in the nucleotide level, which reveals the high number of loci in the species. Therefore, the diversity described in this study is also the reflection of variation in several *DRB* loci, probably generated by gene duplication, not distinguishable from each other.

### Evidence for historical selection

A higher number of nonsynonymous (d*N*) versus synonymous (d*S*) nucleotide substitutions was found for the entire sequence of *DRB* exon 2 gene and in putative ABS, but not in non‐ABS, leading to elevated d*N/*d*S* ratios (Table 3). This scenario, along with the known biological significance of MHC class II genes in peptide presentation for the immune system, is compatible with the hypothesis of positive selection acting in this region. Also, nonsynonymous nucleotide substitutions were more frequently observed in the ABS than in the non‐ABS for all sequences and in each biome separately, as well as higher nucleotide diversity (Table 5, Fig. 6), an indicative of selection acting more strongly on ABS. These findings corroborate the literature on functional importance of these sites (Doherty and Zinkernagel 1975; Hughes and Nei 1988) and support evidence for the correct analogy of ABS positions inferred from humans after Brown et al. (1993) (Fig. 2).

**Table 5:** Average nucleotide and amino acid divergence of individuals within a biome for MHC class II *DRB* exon 2 alleles

Data set	K2P nucleotide distance			Poisson‐corrected amino acid distance		
	All sites	ABS	Non‐ABS	All sites	ABS	Non‐ABS
All Sequences	0.339 (±0.219)	0.302 (±0.071)	0.121 (±0.021)	0.306 (±0.059)	0.594 (±0.260)	0.217 (±0.048)
Atlantic forest	0.105 (±0.017)	0.242 (±0.045)	0.109 (±0.017)	0.270 (±0.043)	0.468 (±0.023)	0.193 (±0.036)
Amazon forest	0.147 (±0.018)	0.248 (±0.046)	0.107 (±0.017)	0.274 (±0.045)	0.481 (±0.132)	0.194 (±0.040)
Cerrado	0.162 (±0.018)	0.275 (±0.049)	0.116 (±0.019)	0.291 (±0.047)	0.506 (±0.133)	0.208 (±0.043)
Caatinga	0.130 (±0.018)	0.233 (±0.048)	0.089 (±0.018)	0.241 (±0.043)	0.446 (±0.120)	0.164 (±0.038)
Pantanal	0.153 (±0.019)	0.254 (±0.049)	0.113 (±0.019)	0.285 (±0.048)	0.472 (±0.139)	0.213 (±0.047)

### *Distribution and diversity of* DRB *alleles among biomes*

Our results show that common and frequent *DRB* alleles are geographically widespread, occurring in all biomes, while other less frequent alleles are restricted to one biome. In fact, all biomes showed some level of private alleles in combination with other rare alleles (Fig. 1). When analyzing the phylogenetic tree (Fig. 7), it is possible to observe that these common widespread alleles are present in all major clades. These alleles are most likely ancient and supposedly very important to the immune response of the species, with probably similar pathogen recognition capabilities, and possibly recognizing a vast array of common antigens. Therefore, they are spread to different lineages and are likely kept in high frequency in all of them by positive selection.

At the same time, several rare alleles were found to be scattered among biomes, and some of them were private to one biome. Nevertheless, the alleles were highly divergent, as evidenced by the phylogenetic tree (Fig. 7) and pairwise genetic distance both in nucleotides and amino acids, and especially in ABS, which showed higher nucleotide diversity and genetic distance than the whole sequence (Table 5, Fig. 6). These parameters are indicative of dissimilar functional properties of these alleles, and the fact that private alleles were found within biomes is considered as evidence that specific alleles may be important in environments which harbor specific pathogens. Curiously, despite Cerrado did not present high frequency of private alleles or allelic richness, it presented the highest genetic divergence against other biomes (both in amino acid and in nucleotide, Table 5), suggesting that specific alleles may play an important role in this different habitat.

Hence, we found different allelic compositions throughout biomes, comprising common, rare, and private alleles. Moreover, rain forest biomes displayed the highest diversity, depicted by higher number of private alleles and higher allelic richness. Between the biomes sampled in this study, the rain forests in fact display higher general diversity among all taxa. Despite the private alleles may not fully reflect biome diversity, as it is dependent on sample size, the cross results from different diversity indicators indicate that the outcome of diversity in rain forests is reliable. As already mentioned, the Amazon and the Atlantic forests are regions with high levels of biodiversity and endemism. Thus, they are also expected to harbor more pathogens than drier or less diverse biomes. This elevated exposure to pathogens is expected to be mirrored in the MHC genetic diversity of the species, as it was observed. On the other hand, our data also controlled for demographic patterns (neutral markers), and the configuration of genetic diversity seemed to be similar to MHC. Thus, the effect of demographic processes such as drift and migration is not negligible in shaping the MHC diversity throughout the species ranges. Studies in humans both on MHC (Prugnolle et al. 2005) and on several genomic SNPs with signs for positive selection (Fumagalli et al. 2011), correlating with pathogen pressure, showed a strong signature of demography despite the observed influence of pathogen‐mediated selection. In fact, Fumagalli et al. (2011) argued that the diversity of the local pathogenic environment could represent a predominant driver of local adaptation, and although background demography usually makes the strongest contribution in explaining the genetic variance among populations, specific alleles could be correlated with a certain pathogenic environment. Studies with nonmodel organisms also showed that demography is important in shaping MHC diversity, even in the presence of clear signatures of selection in this gene family (Alcaide 2010; Miller et al. 2010), with isolation by distance being a common pattern emerging from both microsatellites and MHC. In fact, demography probably does not overcome balancing selection when populations are fragmented and small (Strand et al. 2012). Thus, differences in the spatial distribution of alleles (and genetic differentiation measured by F_ST_, when possible) indicate possible local adaptation to different environments (Ekblom et al. 2007; Miller et al. 2010).

Although our study does not have data about pathogens that interact with our *T. tetradactyla*'s samples, it is possible that the higher MHC diversity found in the rain forest biomes could be the product of a more diverse pathogenic environment (and hence a stronger selective pressure), coupled with the effects of demography, because these biomes display a greater diversification in general than the other sampled landscapes. Larger samples sizes with finer spatial distribution coupled with information about pathogen interaction could help to clarify to what extent (and which) private MHC alleles actually represent local adaptations in the species.

## Conclusions

Our results indicate that MHC variation across lesser anteater populations in different biomes show clear signs of natural selection, as well as probable local adaptation driven by different compositions of pathogens in distinct environments. Rain forest biomes (Amazon and Atlantic forests) show higher overall neutral and adaptive genetic diversity, as expected from known patterns of species diversity in South America biogeography. The higher proportion of MHC private alleles and allelic richness in these biomes points to a different composition of potentially important genetic variation that should be taken into account for future conservation plans.

## Conflict of Interest

No conflict of interests to declare.

## Data Accessibility

- Sequences of *Tamandua tetradactyla* DRB Exon 2 amino acid and nucleotide alleles were deposited in GenBank under the Accession Numbers KP780001 ‐ KP780057.
- Microsatellite sequences of loci were deposited in GenBank under the Accession Numbers KF746177‐KF746185.
- Specific information about samples used in this study can be found in Table S1, Supporting Information.

## Supporting information


**Appendix S1.** Details about SSCP gel preparation.
**Table S1.** List of *Tamandua tetradactyla* samples (*n* = 71) used in this study.
**Table S2.** Fusion primer name and composition (adaptor lib A sequence, internal library key, barcode for individual identification (multiplex identifiers, MIDs) and specific primer sequence (forward: JF1 eV, reverse: YML10) used for 454 pyrosequencing.
