# Supplementary material for: Spatial pattern of adaptive and neutral genetic diversity across different biomes in the lesser anteater (Tamandua tetradactyla)
Source: Ecol Evol. 2015 Oct 15;5(21):4932–48. doi: 10.1002/ece3.1656 (PMC4662318; doi:10.1002/ece3.1656)
Supplement: Supplementary file 1 — Appendix S1. Details about SSCP gel preparation. Table S1. List of Tamandua tetradactyla samples (n = 71) used in this study. Table S2. Fusion primer name and composition (adaptor lib A sequence, internal library key, barcode for individual identification (multiplex identifiers, MIDs) and specific primer sequence (forward: JF1 eV, reverse: YML10) used for 454 pyrosequencing. [file ECE3-5-4932-s001.docx]

**Supporting Information (Clozato et al., Ecology and Evolution)**

**Appendix S1: Details about SSCP gel preparation**

Individual amplicons were screened by SSCP analysis (Orita *et al*. 1989), a method that can detect variants separated by only a single base difference (Sunnucks *et al*. 2000). For denaturation, 2–4 µl of PCR products were mixed with 6 µl of loading dye (30% glycerol, 0.25% bromophenol blue, 0.25% xylene cyanol), heated at 95ºC for 5’ and placed on ice for 5’. This mix was loaded on 15% non-denaturing polyacrylamide gels (ETC, Germany) and run on a horizontal cooling electrophoresis system (Amersham Pharmacia, Germany) with the following conditions: 200 V, 10 mA, 10 W for 20’ followed by 450 V, 30 mA, 20 W for 3:45’ as well as 4:30’ at a constant temperature of 10 ºC. Gels were fixed and silver-stained using PlusOne DNA Silver Staining Kit (Amersham Pharmacia) following the manufacturer’s recommendations. Distinctive single-strand bands were excised from the gel, eluted in 30 µl TBE buffer and incubated for at least 3 hours. Subsequent reamplifications followed as described in the Material and Methods section.

*References*

Orita M, Iwahana H, Kanazawa H, Hayashi K, Sekiya T (1989) Detection of polymorphisms of human DNA by gel electrophoresis as single-strand conformation polymorphisms. *Proc Natl Acad Sci* *U S A.* **86**, 2766–2770.

Sunnucks P, Wilson ACC, Beheregaray LB, Zenger K, French J, Taylor AC (2000) SSCP is not so difficult: the application and utility of single-stranded conformation polymorphism in evolutionary biology and molecular ecology. *Mol Ecol* **9**, 1699– 1710.

**Table S1** - List of *Tamandua tetradactyla* samples (n=71) used in this study. Sample ID, biome of origin (according to IBGE (<http://www.ibge.gov.br/home>): Atlantic Forest - AF, Amazon Forest - AM, Caatinga - CA, Cerrado – CE and Pantanal - PT), country, Brazilian federal state abbreviation, locality/municipality, and reference institution where the voucher specimen, tissue or DNA is stored (Laboratório de Biologia Evolutiva e Conservação de Vertebrados, São Paulo – LABEC; Laboratório de Biodiversidade e Evolução Molecular, Belo Horizonte – LBEM; Museu de Biologia Professor Mello Leitão, Santa Teresa – MBML; Universidade Federal de Rondônia, Boa Vista – UNIR; Laboratório de Biologia Genômica e Molecular, Porto Alegre – LBGM), latitude and longitude.

| Sample ID | Biome | Country | Federal State^1^ | Locality/Municipality | Reference institution | Longitude | Latidude |
| --- | --- | --- | --- | --- | --- | --- | --- |
| M0659 | AF | Brazil | MG | Belo Horizonte | LBEM | 19° 55' 00" S | 043° 56' 00"W |
| M0714 | AF | Brazil | MG | Belo Horizonte | LBEM | 19° 55' 00" S | 043° 56' 00" W |
| M0716 | AF | Brazil | MG | Sto Antônio do Amparo | LBEM | 20° 57' 00" S | 44° 55' 00" W |
| M0661 | AF | Brazil | MG | Abre Campo | LBEM | 20° 18' 00" S | 42° 29' 00" W |
| M0666 | AF | Brazil | MG | Acaiaca | LBEM | 20° 21' 00" S | 43° 09' 00" W |
| M0703 | AF | Brazil | BA | Una | LBEM | 39º 2' 3" S | 15º 11' 36" W |
| M0691 | AF | Brazil | SP | Bauru | LBEM | 22° 19' 00" S | 49° 04' 00" W |
| M0694 | AF | Brazil | SP | Bauru | LBEM | 22° 19' 00" S | 49° 04' 00" W |
| M0973 | AF | Brazil | PR | Telemaco Borba | LBEM | 24° 21' 00" S | 50° 37' 00" W |
| M0974 | AF | Brazil | PR | Telemaco Borba | LBEM | 24° 21' 00" S | 50° 37' 00" W |
| M0975 | AF | Brazil | PR | Telemaco Borba | LBEM | 24° 21' 00" S | 50° 37' 00" W |
| TTGI01 | AF | Brazil | MG | Resplendor | LBEM | 19° 20' 00" S | 41° 15' 00" W |
| TTCA01 | AF | Brazil | MG | Barão de Cocais | LBEM | 19° 56' 00" S | 043° 28' 00" W |
| TTNA01 | AF | Brazil | MG | Belo Horizonte | LBEM | 19° 56' 50"S | 43° 54' 11" W |
| MBML2453 | AF | Brazil | ES | Santa Teresa | MBML | 20° 23' 24” S | 40° 29' 45” W |
| TMSJBV | AF | Brazil | SP | São João da Boa Vista | LABEC | 21º 58' 09" S | 46º 47' 53" W |
| TRS01 | AF | Brazil | RS | Vacaria | LABEC | 28° 30' 00" S | 050° 56' 00" W |
| TMIguape | AF | Brazil | SP | Iguape | LABEC | 24° 43' 00" S | 47° 33' 00" W |
| TMPauloA | AF | Brazil | SP | Caraguatatuba | LABEC | 23° 37' 00" S | 45° 25' 00" W |
| T1AL | AF | Brazil | AL | Maceió | LABEC | 09° 40' 00" S | 35° 43' 00" W |
| T2AL | AF | Brazil | AL | Maceió | LABEC | 09° 40' 00" S | 35° 43' 00" W |
| T3AL | AF | Brazil | AL | Rio Largo | LABEC | 09° 30' 15" S | 35° 49' 43" O |
| T4AL | AF | Brazil | AL | Maceió | LABEC | 09° 36’ 10” S | 35° 45’ 86” W |
| T5PE | AF | Brazil | PE | Recife | LABEC | 08° 03' 00" S | 34° 54' 00" W |
| T6PE | AF | Brazil | PE | Recife | LABEC | 08° 03' 00" S | 34° 54' 00" W |
| T7PE | AF | Brazil | PE | Goiana | LABEC | 07° 33' 00" S | 34° 59' 00" W |
| T8PE | AF | Brazil | PE | Igarassu | LABEC | 07° 50' 00" S | 34° 54' 00" W |
| T10PE | AF | Brazil | PE | Igarassu | LABEC | 07º 46’ 97” S | 35º 00’ 74” W |
| T11PB | AF | Brazil | PB | João Pessoa | LABEC | 07° 07' 00" S | 34° 52' 00" W |
| T12PB | AF | Brazil | PB | João Pessoa | LABEC | 07° 07' 00" S | 34° 52' 00" W |
| Btte010 | AF | Brazil | RS | Vacaria | LBGM | 28° 30' 00" S | 50° 56' 00" W |
| TTAMZ01 | AM | Brazil | PA | Marabá | LABEC | 05° 21' 00" S | 49° 07' 00" W |
| TMPar | AM | Brazil | AM | Parintins | LABEC | 02° 36' 00" S | 56° 44' 00" W |
| TMAM | AM | Brazil | PA | Oriximiná | LABEC | 01° 45' 55" S | 55° 51' 50" W |
| UFROM302 | AM | Brazil | RO | Porto Velho | UNIR | 08° 46' 00" S | 63° 54' 00" W |
| UFROM303 | AM | Brazil | RO | Porto Velho | UNIR | 08° 46' 00" S | 63° 54' 00" W |
| TTMT1* | AM | Brazil | MT | Pontes e Lacerda | LABEC | 15 ° 49' 43" S | 57°54' 42" W |
| TTMT2* | AM | Brazil | MT | Pontes e Lacerda | LABEC | 15° 42' 00" S | 58°42' 37" W |
| TTMT3 | AM | Brazil | MT | Pontes e Lacerda | LABEC | 15°24' 48" S | 57° 07' 54" W |
| TMPeru1 | AM | Peru | - | - | LABEC | 10° 39' S** | 71° 38' W** |
| TMPeru2 | AM | Peru | - | - | LABEC | 10° 39' S** | 71° 38' W** |
| TMPeru3* | AM | Peru | - | - | LABEC | 10° 39' S** | 71° 38' W** |
| 13H47 | AM | French Guyana | - | Kourou | LABEC | 05° 09' 00" N | 52° 39' 00" W |
| M0658 | CE | Brazil | MG | Mariana | LBEM | 16° 41' 00" S | 45° 02' 00" W |
| M0667 | CE | Brazil | GO | São Simão | LBEM | 18º 49' 33" S | 50º 30' 21" W |
| TTTA01 | CE | Brazil | MT | Nova Xavantina | LBEM | 14° 27' 51" S | 52° 21' 41" W |
| TTTA02 | CE | Brazil | MT | Nova Xavantina | LBEM | 14° 27' 51" S | 52° 21' 41" W |
| TTJE01 | CE | Brazil | GO | Catalão | LBEM | 18° 10' 00" S | 47° 57' 00" W |
| TTPAN8 | CE | Brazil | MS | Paranaíba | LABEC | 19° 40' 00" S | 51° 11' 00" W |
| TTPAN9 | CE | Brazil | MS | Paranaíba | LABEC | 19° 40' 00" S | 51° 11' 00" W |
| TMUnimat | CE | Brazil | MT | Nova Xavantina | LABEC | 14° 40' 22” S | 52° 21' 10” W |
| Btte008 | CE | Brazil | MS | Anaurilândia | LBGM | 22° 03' 00" S | 52° 45' 00" W |
| Btte006 | CE | Brazil | MS | Bataguaçu | LBGM | 21° 42' 50" S | 52° 25' 19" W |
| M0688 | CE | Brazil | SP | Lorena | LBEM | 22° 44' 00" S | 45° 08' 00" W |
| TTBA01 | CE | Brazil | BA | Luís Eduardo Magalhães | LABEC | 05° 92' 83"S | 42° 69' 82" W |
| M0692 | CE | Brazil | SP | Lorena | LBEM | 22° 19' 00" S | 49° 04' 00" W |
| TOC015 | CE | Brazil | MA | Imperatriz | LABEC | 05° 32' 00" S | 47° 29' 00" W |
| TOC065 | CE | Brazil | MA | Imperatriz | LABEC | 05° 32' 00" S | 47° 29' 00" W |
| TOC001 | CE | Brazil | MA | Imperatriz | LABEC | 05° 32' 00" S | 47° 29' 00" W |
| T13CE | CA | Brazil | CE | Fortaleza | LABEC | 03° 43' 00" S | 38° 30' 00" W |
| T16CE | CA | Brazil | CE | Ipiapaba | LABEC | 05° 03' 00" S | 40° 55' 00" W |
| T17CE | CA | Brazil | CE | Sobral | LABEC | 03° 42' 00" S | 40° 21' 00" W |
| TTPAN1 | PAN | Brazil | MS | Aquidauana | LABEC | 20º 30’ 10” S | 55º 51’ 52” W |
| TTPAN2 | PAN | Brazil | MS | Aquidauana | LABEC | 20º 29’ 55” S | 55º 55’ 39” W |
| TTPAN3 | PAN | Brazil | MS | Aquidauana | LABEC | 20º 06’ 03” S | 55º 58’ 52” W |
| TTPAN4 | PAN | Brazil | MS | Aquidauana | LABEC | 20º 05’ 42” S | 55º 57’ 53” W |
| TTPAN5 | PAN | Brazil | MS | Aquidauana | LABEC | 20º 17’ 30” S | 56º 17’ 9” W |
| TTPAN7 | PAN | Brazil | MS | Aquidauana | LABEC | 20º 05’ 42” S | 55º 57’ 53” W |
| TTPAN11* | PAN | Brazil | MS | Aquidauana | LABEC | 19° 40' 00" S | 51° 11' 00" W |
| TTPAN12* | PAN | Brazil | MS | Aquidauana | LABEC | 19° 16' 47" S | 57° 34' 25" W |
| TTPAN13* | PAN | Brazil | MS | Aquidauana | LABEC | 19° 16' 47" S | 57° 34' 25" W |

^1^Names of Brazilian federal states, in order of appearance: Minas Gerais (MG), Bahia (BA), São Paulo (SP), Paraná (PR), Espírito Santo (ES), Rio Grande do Sul (RS), Alagoas (AL), Pernambuco (PE), Paraíba (PB), Pará (PA), Amazonas (AM), Rondônia (RO), Goiás (GO), Mato Grosso (MT), Mato Grosso do Sul (MS), Ceará (CE).

*Samples that did not yield enough coverage through pyrosequencing, and were excluded from further analyses.

** Approximate location.

**Table S2** – Fusion primer name and composition (adaptor Lib-A sequence, internal library key, barcode for individual identification (multiplex identifiers, MIDs) and specific primer sequence (forward: JF1eV, reverse: YML10) used for 454 pyrosequencing.

| **Fusion Primer Name** | **Adaptor** | **Key** | **Barcode (MID)** | **Specific Primer Sequence** |
| --- | --- | --- | --- | --- |
| **Forward** |  |  |  |  |
| SS_MHC_JF1eV_LibA_A001 | CGTATCGCCTCCCTCGCGCCA | TCAG | ACGAGTGCGT | GAGTGTCATTTYGAGAACGGGACSGAG |
| SS_MHC_JF1eV_LibA_A002 | CGTATCGCCTCCCTCGCGCCA | TCAG | AGACGCACTC | GAGTGTCATTTYGAGAACGGGACSGAG |
| SS_MHC_JF1eV_LibA_A003 | CGTATCGCCTCCCTCGCGCCA | TCAG | CGTGTCTCTA | GAGTGTCATTTYGAGAACGGGACSGAG |
| SS_MHC_JF1eV_LibA_A004 | CGTATCGCCTCCCTCGCGCCA | TCAG | CTCGCGTGTC | GAGTGTCATTTYGAGAACGGGACSGAG |
| SS_MHC_JF1eV_LibA_A005 | CGTATCGCCTCCCTCGCGCCA | TCAG | TACAGATCGT | GAGTGTCATTTYGAGAACGGGACSGAG |
| SS_MHC_JF1eV_LibA_A006 | CGTATCGCCTCCCTCGCGCCA | TCAG | TGATACGTCT | GAGTGTCATTTYGAGAACGGGACSGAG |
| SS_MHC_JF1eV_LibA_A007 | CGTATCGCCTCCCTCGCGCCA | TCAG | CATACTCTAC | GAGTGTCATTTYGAGAACGGGACSGAG |
| SS_MHC_JF1eV_LibA_A008 | CGTATCGCCTCCCTCGCGCCA | TCAG | CGAGAGATAC | GAGTGTCATTTYGAGAACGGGACSGAG |
| SS_MHC_JF1eV_LibA_A009 | CGTATCGCCTCCCTCGCGCCA | TCAG | ATACGACGTA | GAGTGTCATTTYGAGAACGGGACSGAG |
| SS_MHC_JF1eV_LibA_A010 | CGTATCGCCTCCCTCGCGCCA | TCAG | TCTGTCTCGC | GAGTGTCATTTYGAGAACGGGACSGAG |
| SS_MHC_JF1eV_LibA_A011 | CGTATCGCCTCCCTCGCGCCA | TCAG | CGACACTATC | GAGTGTCATTTYGAGAACGGGACSGAG |
| **Reverse** |  |  |  |  |
| SS_MHC_YML10_LibA_B001 | CTATGCGCCTTGCCAGCCCGC | TCAG | TCTCTATGCG | TCGCCGCTGCACTGTGAACGTCTC |
| SS_MHC_YML10_LibA_B002 | CTATGCGCCTTGCCAGCCCGC | TCAG | TCACGCGAGA | TCGCCGCTGCACTGTGAACGTCTC |
| SS_MHC_YML10_LibA_B003 | CTATGCGCCTTGCCAGCCCGC | TCAG | TAGAGACGAG | TCGCCGCTGCACTGTGAACGTCTC |
| SS_MHC_YML10_LibA_B004 | CTATGCGCCTTGCCAGCCCGC | TCAG | TCGTCGCTCG | TCGCCGCTGCACTGTGAACGTCTC |
| SS_MHC_YML10_LibA_B005 | CTATGCGCCTTGCCAGCCCGC | TCAG | ATAGATAGAC | TCGCCGCTGCACTGTGAACGTCTC |
| SS_MHC_YML10_LibA_B006 | CTATGCGCCTTGCCAGCCCGC | TCAG | TAGTCGCATA | TCGCCGCTGCACTGTGAACGTCTC |
| SS_MHC_YML10_LibA_B007 | CTATGCGCCTTGCCAGCCCGC | TCAG | TGTACTACTC | TCGCCGCTGCACTGTGAACGTCTC |
| SS_MHC_YML10_LibA_B008 | CTATGCGCCTTGCCAGCCCGC | TCAG | ACGACTACAG | TCGCCGCTGCACTGTGAACGTCTC |
| SS_MHC_YML10_LibA_B009 | CTATGCGCCTTGCCAGCCCGC | TCAG | CGTAGACTAG | TCGCCGCTGCACTGTGAACGTCTC |
| SS_MHC_YML10_LibA_B010 | CTATGCGCCTTGCCAGCCCGC | TCAG | CTATAGCGTA | TCGCCGCTGCACTGTGAACGTCTC |
